# On the Relationship Between White Matter Structure and Subjective Pain. Lessons From an Acute Surgical Pain Model

**DOI:** 10.3389/fnhum.2020.558703

**Published:** 2020-11-24

**Authors:** Laura Torrecillas-Martínez, Andrés Catena, Francisco O'Valle, César Solano-Galvis, Miguel Padial-Molina, Pablo Galindo-Moreno

**Affiliations:** ^1^Department of Oral Surgery and Implant Dentistry, School of Dentistry, University of Granada, Granada, Spain; ^2^Mind, Brain and Behavior Research Center (CIMCYC), University of Granada, Granada, Spain; ^3^Department of Pathology, School of Medicine and Instituto de Biopatología y Medicina Reparativa, University of Granada, Granada, Spain

**Keywords:** analgesia - trends, anesthesia, acute pain, tractography, fractional anisotropy, white matter structure

## Abstract

**Background:** Pain has been associated with structural changes of the brain. However, evidence regarding white matter changes in response to acute pain protocols is still scarce. In the present study, we assess the existence of differences in brain white matter related to pain intensity reported by patients undergoing surgical removal of a mandibular impacted third molar using diffusion tensor imaging (DTI) analysis.

**Methods:** 30 participants reported their subjective pain using a visual analog scale at three postsurgical stages: under anesthesia, in pain, and after the administration of an analgesic. The diffusion data were acquired prior to surgery.

**Results:** DTI analysis yielded significant positive associations of fractional anisotropy in white matter areas related to pain processing (corticospinal tract, corona radiata, corpus callosum) with the differences in pain between the three postsurgery stages. Extent and location of these associations depended on the magnitude of the subjective pain differences. Tractography analysis indicated that some pain–tract associations are significant only when pain stage is involved in the contrast (posterior corona radiata), while others (middle cerebellar peduncle, pontine crossing) are only when anesthesia is involved in the contrast.

**Conclusions:** The association of white matter fractional anisotropy and connectivity, measured before the pain stages, with subjective pain depends on the magnitude of the differences in pain scores.

## Introduction

Chronic pain has been associated with structural changes of the brain, including prefrontal, somatosensory and occipital areas and subcortical nuclei (Cauda et al., 2014; Kuner and Flor, 2017; Yuan et al., 2017; Shokouhi et al., 2018). Gray matter structural changes have been observed in chronic pain conditions (Diaz-Piedra et al., 2016; Jia and Yu, 2017; Wang et al., 2017) as well as changes in white matter (Jutzeler et al., 2016; Tian et al., 2016; Hotta et al., 2017; Malfliet et al., 2017; Zhong et al., 2018).

Brain morphometric alterations have been documented also in acute pain conditions (Emerson et al., 2014), indicating that the volume of some pain-processing brain regions can be affected by them (Elsenbruch et al., 2014), even when the strength of these associations is rather modest (4–5% Stankewitz et al., 2013) or nonsignificant (i.e., pressure pain threshold Ruscheweyh et al., 2018). Brain functional changes have been reported after repetitive pain stimulation (Stankewitz et al., 2013), especially in subgenual anterior cingulate. These changes last up to 1 year (Bingel et al., 2008), indicating that habituation to painful stimuli is key in pain perception (Ginzburg et al., 2015). Repetitive pain stimulation changes gray matter concentration of pain-related areas in individuals who do not show habituation (Stankewitz et al., 2013). However, gray matter changes tend to recede when the nociceptive input stops (Teutsch et al., 2008), or the chronic pain is relieved (Lewis et al., 2018). This suggests that acute pain can be associated with brain structural changes, and the association between brain structure and pain can be observed independently of contextual factors or level of pain experienced. Evidence regarding white matter changes in response to acute pain protocols is scarce, although some reports of abnormal regional fractional anisotropy (FA) and structural connectivity, measured by tractography, in the prefrontal cortex may underlie the transition (Chapman and Vierck, 2017) from acute to chronic pain (Vachon-Presseau et al., 2016).

The present study aims to determine whether white matter integrity and structure are associated with acute pain, and more specifically, whether variations in pain intensity are linked to fractional anisotropy and white matter connectivity. We used an orofacial pain model, the extraction of mandibular impacted third molars. This pain lasts for hours, but its intensity changes in accordance with three typical stages: (1) Anesthesia, applied before the surgery and lasting approx 3 h, (2) pain, cessation of the anesthetic agent, and (3) analgesia, postoperative medication prescribed. Patients were asked to indicate their pain intensity at the three stages (hereafter, subjective pain). We hypothesize that subjective pain will differ between stages, so that pain is greater than anesthesia, pain is greater than analgesia, and anesthesia is greater than analgesia, but the associations of these differences in subjective pain and white matter structure must remain constant, as there is no time for structural changes to happen, as our protocol lasted 4 h, and there is no scientific literature pointing out to changes in white or gray matter in such a sort of period of time. Furthermore, if changes were observed this will indicate that these associations reflect the recruitment of more (or less) structures to process pain, rather than a change in the nature of the pain–brain structure relationships.

## Materials and Methods

### Participants

This prospective study was conducted after approval by the University of Granada Ethics Committee on Human Research (n° 877) and in accordance with the Declaration of Helsinki for treatment of experimental human subjects (Williams, 2008), and it adheres to the applicable STROBE guidelines. Subjects received detailed information about the surgical and scanning procedures and they or their legal representatives provided written informed consent.

A total of 30 subjects (23 women) were recruited for the present study (mean age, 21.83 years, range 18–32 years). Participants were healthy and were included only if they had a mandible impacted third molar which needed surgical procedure to be removed, but they were completely free of pain at the start of the study. All molars were located in the mandible (50% left). Participants were recruited from the pool of patients/students of the School of Dentistry of the University of Granada and were part of a larger study on the effects of oral surgery on brain structure and function. Power analysis (α = 0.05, power = 0.9, and partial R-square = 0.3) on subjective measures suggested a total sample size of 27 participants. We added three more participants, assuming a dropout of 10%.

### Surgical Procedure and Pain Measurement

The third molars were extracted by an expert oral surgeon (PGM) following a standardized surgical procedure performed at the School of Dentistry at University of Granada. In short, an inferior alveolar and buccal nerve block was given using 1.8-mL carpule of 2% articaine with adrenaline 1:100,000. Surgical access was routinely achieved buccally through a triangular flap. Bone removal around the tooth was then performed with a round bur on a straight hand-piece under continuous irrigation. Crown and/or roots were sectioned when necessary. After extraction, the socket was inspected and the flap was sutured back by 2–4 interrupted stitches using a 3–0 silk suture. Gauze impregnated with either chlorhexidine or hyaluronic acid was applied over the socket, and the usual post extraction instructions were given written to the patient. All the patients received routinely the following postoperative medication: amoxicillin/clavulanic acid 2 g twice a day and dexketoprofen 25 mg three times a day for 7 days; metamizole 565 mg was given as a rescue analgesic. The duration of surgery (from the incision to the extraction) and the duration of the anesthetic agent in minutes, among others, were recorded.

Patients were asked to report their subjective pain using a visual analog Likert-type scale (hereafter, subjective pain), in which 0 meant “no pain at all” and 10 meant “worst pain imaginable,” at three different times: anesthesia, pain, and analgesia. The anesthesia scoring was taken 30 min after the surgical procedure, and patients were still under anesthesia effects (anesthesia stage). The second measure was taken either when local anesthetic ceased its function, as reported by the participant, or 3 h after the extraction (pain stage). At this moment, patients were supposed to be at the pain peek, caused by the surgical trauma provoked by the dental surgery. The length of these two intervals (30 min and 3 h.) was selected according to Senes et al. (2015), who reported anesthesia durations around 3 h. Then, all participants were given an analgesic (Metamizole 565 mg, Laboratorios Normon S.A, Spain) in order to reduce their pain. Thirty minutes after the analgesic, the last measure of pain was taken (analgesia stage). This interval was based on Schmieder et al. (1993) metamizole onset time 10.9 (±5.8) min.

### DTI Scanning

Participants were scanned before the surgery using a DTI diffusion scheme. The diffusion images were acquired on a SIEMENS Trio Tim scanner located at the Mind, Brain and Behavior Research Center of the University of Granada using a 2D EPI diffusion sequence, with TE = 90 ms, and TR = 3,300 ms. A HARDI scheme was used, and a total of 30 diffusion sampling directions were acquired, three times each. The b-value was 1,000 s/mm^2^. A non-diffusion scan was obtained with b-value = 0 s/mm^2^. The in-plane resolution was 1.8 mm. The slice thickness was 5.2 mm. A T1-weighted anatomical scan was also obtained for each participant using a MPRAGE sequence (TR = 1,900 ms; TE = 2.52 ms; flip angle = 9°, voxel size = 1 × 1 × 1 mm^3^; FOV = 256 × 256 mm^2^; matrix size = 256 × 256, 176 slices). The average interval between session and third molar surgery was 23.6 (±42.41) days.

### White Matter Analysis

#### White Matter Integrity

Diffusion tensor imaging analysis was carried out using the FB Software Library (FSL, https://fsl.fb.ox.ac.uk/fsl Jenkinson et al., 2012), which includes eddy current and motion correction, brain mask extraction (BET v2.1 Smith, 2002), reconstruction of the diffusion tensors (DTIFIT), and computation of fractional anisotropy (FA). Mean diffusivity (MD) was obtained as the average of the three eigenvalues (L1, L2, L3). Axial diffusivity (AD) was obtained as the first eigenvalue (L1). Voxel-wise Tract-Based Spatial Statistics (TBSS), a part of the FSL software, was used to examine the association of the differences between subjective pain intensities at the three postsurgical stages (pain–anesthesia, pain–analgesia, and anesthesia–analgesia) and FA, MD, and AD. In short, all the FA volumes were nonlinearly registered to the FB58-FA template (https://fsl.fb.ox.ac.uk/fsl/fslwiki/data/FB58_FA.html) and aligned to the Montreal Neurological Institute space. A FA skeleton was created by first averaging all the FA volumes and thinning the average, with a 0.2 FA threshold. The aligned FA volume of each participant was projected onto this FA skeleton and submitted to the statistical analysis. A randomization procedure (2,000 random permutations, FSL's randomize software) was used to perform the multiple regression analysis, in which the variables of interest were the between-stage differences in pain intensity, and the nuisances were age and gender. The results were corrected using threshold-free cluster enhancement (TFCE) correction for multiple comparisons (family-wise error rate *p* = 0.05). The same analyses were applied to the MD and AD volumes, after aligning them into the MNI space and projecting them onto the mean FA skeleton using the non-FA script included in FSL. No significant effects were observed in MD and AD.

#### Connectometry Analysis

The diffusion data were eddy and motion corrected and averaged across repetitions to improve the signal-to-noise ratio. The volumes were reconstructed in MNI space using q-space diffeomorphic reconstruction (Yeh et al., 2011) to obtain the spin distribution function (SDF) (Yeh et al., 2010). The diffusion sampling length ratio was 1.25. The output resolution was 2 mm.

Diffusion magnetic resonance imaging (MRI) connectometry (Yeh et al., 2016) was used to study the association of pain intensity with white matter spin distribution function. The SDF measures the density of water diffusion, instead of the speed of water diffusion, as diffusivity measures do. Connectometry analysis tracks de association, so that the first identifies voxels with a high association and then tracks along the fiber direction to determine the consecutive fiber segments that also show that association. We used a multiple-regression analysis, in which the predictors of interest were the between-stage differences in pain intensity: anesthesia–pain, pain–analgesia, and anesthesia–analgesia. The variables of no interest were gender and age. One t-tresholds (3.02) were used to select local connectomes to provide high sensitivity (lower threshold) and high specificity (higher threshold). A deterministic fiber-tracking algorithm, implemented in DSI_Studio (http://dsi-studio.labsolver.org/; Yeh et al., 2013), was used to estimate fiber directions in whole-brain regions. All tracks generated from bootstrap resampling were included. The length threshold to select tracks was 40 mm. The seeding density was 20 seeds/mm^3^. We used a *p* < 0.05 false discovery ratio (FDR) to control for the multiple-comparison problem. FDR was estimated in a total of 2,000 random permutations to determine the null distribution of track length. All the analyses were done using DSI Studio.

## Results

Subjective pain intensities were higher at the pain stage than at the anesthesia [averages = 4.3 and 5.5, respectively, *t*(29) = 4.15, *p* < 0.001] and analgesia [average= 2.25, *t*(29) = 6.81, *p* < 0.001] stages and were higher at anesthesia than at analgesia [*t*(29) = 4.41, *p* < 0.001].

### Diffusivity

**Figure 1:**
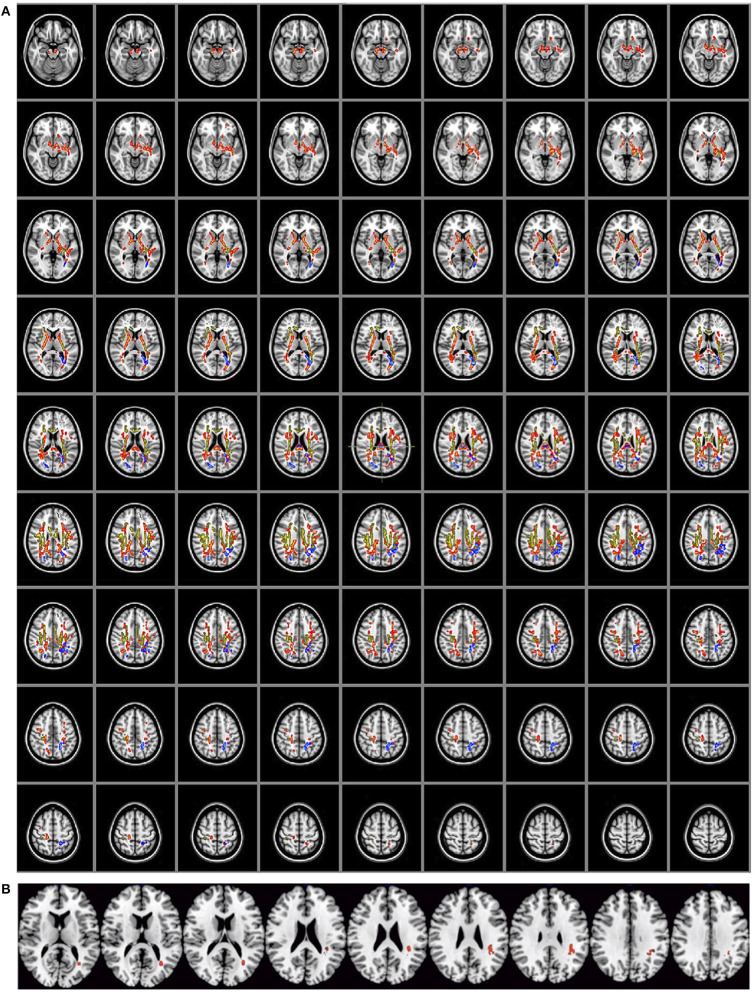
**(A)** Diffusivity (*n* = 30). Significant positive associations of fractional anisotropy with differences in pain intensities between the three stages: pain–anesthesia (blue), pain–analgesia (red), and anesthesia–analgesia (yellow). **(B)** Areas (right superior longitudinal fasciculus) in which the three associations overlap.

The positive associations between FA and the difference between pain intensities (pain–anesthesia, pain–analgesia, and anesthesia–analgesia) are displayed in Table 1 and Figure 1. No negative associations were observed. The set of significant tracts, according to the Juelich Histological atlas, included in FSL, embraces posterior parts of corpus callosum (splenium), and left and right corticospinal tract. Figure 1A shows that the location of these tracts partially overlaps (Figure 1B) but also that there are unique areas associated with each one of the subjective pain difference (Table 1). No differences were observed as a function of the surgery location (all corrected *p* > 0.22). Moreover, the number of significant voxels seems to depend on the size of the difference between postsurgical stages, that is, between subjective pain intensities (Table 1). No differences were observed for MD and AD.

**Table 1:** Fractional anisotropy.

**Contrast**	**Cluster #**	**Cluster peak tract**	***k***	**X**	**Y**	**Z**	**Difference pain intensity**
Pain–anesthesia	1	Callosal body/anterior intra-parietal sulcus L	1,153	−20	−48	46	
	2	Optic radiation L/callosal body	842	−31	−67	13	
	3	Optic radiation R/callosal body	343	25	−77	24	
	4	Callosal body	299	−2	−28	22	
	Global		2,637				1.20
Pain–analgesia	1	Corticospinal tract L/callosal body/primary somatosensory cortex BA3a	9,743	−31	−67	3	
	2	Corticospinal tract R/callosal body	7,739	37	−38	22	
	3	Callosal body	372	−1	−27	23	
	4	Superior longitudinal fasciculus L	291	−32	4	33	
	Global		1,8143				3.25
Anesthesia–analgesia	1	Corticospinal tract R/primary somatosensory cortex BA3a R	2,525	43	−10	27	
	2	Corticospinal tract L/callosal Body/cingulum L	1,802	−27	−24	22	
	3	Fibers adjacent to anterior intra-parietal sulcus	385	−37	−42	20	
	4	Callosal body	208	−32	−47	14	
	5	Corticospinal tract L/primary somatosensory cortex BA3a	141	−36	−14	25	
	6	Callosal body/cingulum R	88	10	7	27	
	Global		5,149				2.05

*k, number of significant voxels in the cluster. Global, total number of significant voxels in the contrast. X, Y, and Z are the Montreal Neurological Institute (MNI) coordinates for the significance peaks*.

*White matter tracts significantly related to differences in subjective pain intensity between the three postsurgery stages*.

### Connectivity

**Figure 2:**
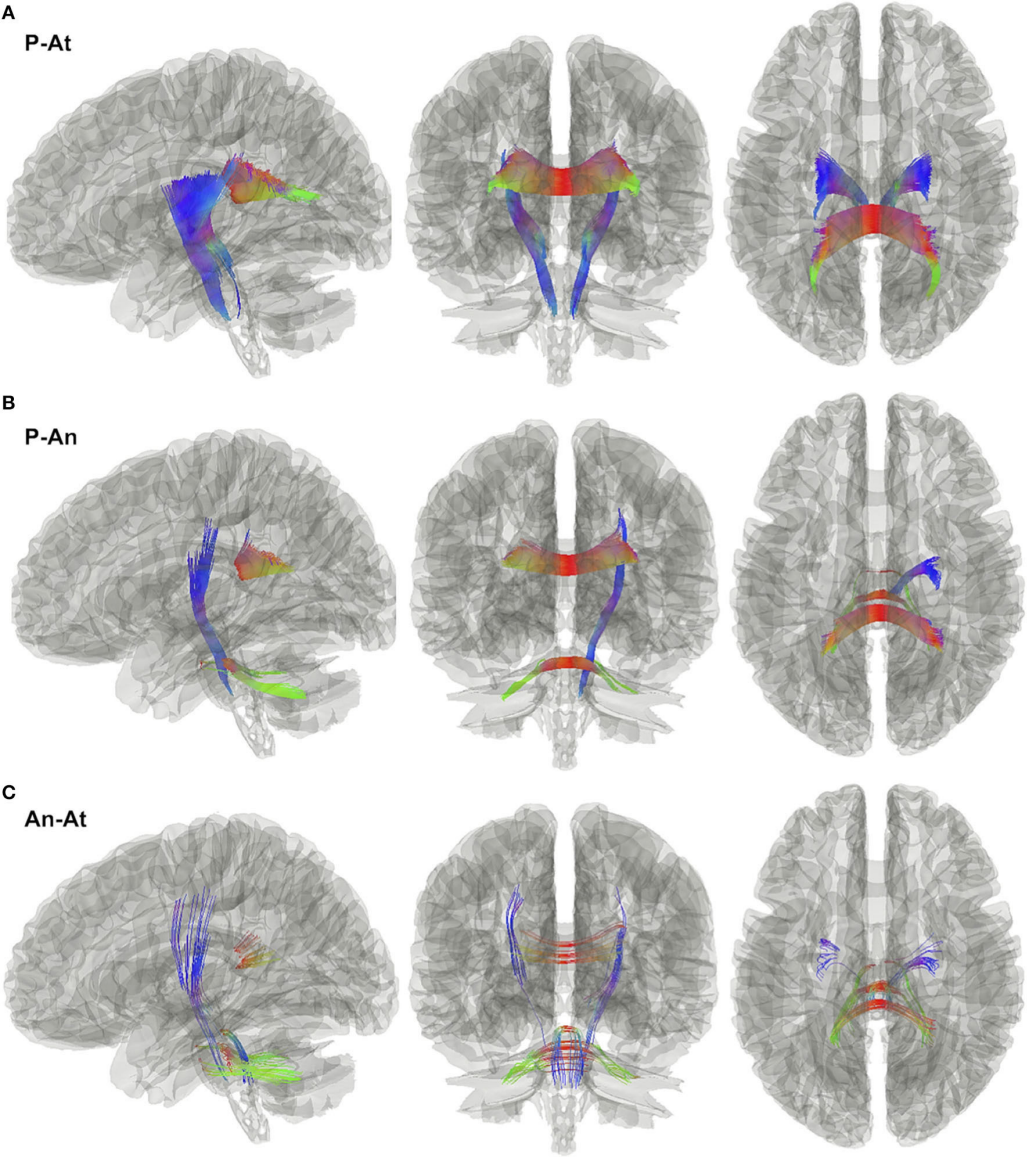
White matter tracks (*n* = 30) positively associated with subjective pain intensity differences between the three postsurgical stages. **(A)** Pain–anesthesia, **(B)** pain–analgesia, and **(C)** anesthesia–analgesia at the 2.5 threshold.

The connectometry analysis (Table 2, Figure 2) identified significant increased tracks connectivity (FDR corrected *p* < 0.05) related to pain–anesthesia, to pain–analgesia, and anesthesia–analgesia subjective pain differences. We observed significant associations in cerebral peduncles, corticospinal tracts, medial lemniscus, splenium of the corpus callosum, and posterior limb of the internal capsule. Middle cerebellar peduncle and superior corona radiata are involved only when analgesia postsurgical stage enters into the contrast (pain–analgesia and anesthesia–analgesia). No tracts showed significant decreased connectivity related to any of the differences between subjective pain intensities.

**Table 2:** Connectivity analysis.

**P-At**	**P-An**	**At-An**
Corticospinal tract R/L	Corticospinal tract R	Corticospinal tract R/L
Cerebral peduncle R/L	Cerebral Peduncle R	Cerebral peduncle R/L
Medial lemniscus R/L		Medial lemniscus R/L
	Middle cerebellar peduncle	Middle cerebellar peduncle
Posterior limb of the internal capsule R/L	Posterior limb of the internal capsule R	Posterior limb of the internal capsule R/L
Splenium	Splenium	Splenium
	Pontine crossing tract	Pontine crossing tract
Posterior corona radiata R/L	Posterior corona radiata R	
Superior corona radiata R/L	Superior corona radiata R	Superior corona radiata R/L

*P-At, pain–anesthesia; P-An, pain–analgesia; At-An, anesthesia–analgesia; R/L, right/left hemisphere. Tracts identified using the JHU ICBM-DTI-81 White-Matter Labels atlas*.

*Significant tracts positively related to differential pain scores*.

## Discussion

Two main results have been observed in this study: first, the association of pain with white matter integrity and connectivity, and second, the dependency of these associations of the level of pain experienced during the protocol. The causal role of pain on brain structure has been a matter of intense research during the last decades. Nowadays, data on brain gray matter volume and white matter integrity suggest that pain is strongly associated with brain morphology changes and plasticity (Chapman and Vierck, 2017; Kuner and Flor, 2017). Most of these data have been observed when using a between-subject approach, in which a chronic pain condition is commonly compared with a non-pain one. Here, we used a postsurgical pain model (third impacted molar) in which pain changes from the anesthesia stage, immediately posterior to the surgery, followed by a short pain stage, to the final analgesia stage. These pain changes happen in such a short time interval (a few hours) that changes in brain structure are extremely improbable, and, therefore, a single pre-surgery DTI scan allows us to ascertain, first, whether acute pain is associated with white matter integrity and connectivity, and, second, whether these associations depended on the level of experienced acute pain.

The white matter tracts we have observed in the present study are involved in the processing of pain, including orofacial one (Moayedi et al., 2012; Wang et al., 2017). The medial lemniscus pathway, specially its medial border, conveys information from the principal sensory nucleus and the spinal nucleus, whose afferents are originated in the oral cavity, and play a role in the transmission of propioceptive information, including pain after mechanical stimulation (Henssen et al., 2016). Similarly, associations of pain with sensorimotor tracts can be related to the differences in the inputs originated in the oral cavity (Moayedi et al., 2012) between the three postsurgery stages that may influence the perceived pain. However, as DTI is unable to discriminate ascending from descending pathways, we cannot discard that motor commands to the orofacial region can be a function of the postsurgery stage, which again influences the subjective experience of pain. The cerebellum has also a role in pain perception (Bocci et al., 2015), probably because nociceptive information is conveyed to the Purkinje cells either by the spino-olivocerebellar or by the spino-pontocerebellar pathways (Baumann et al., 2015). Moreover, the fact that pontine crossing tract (the entry points of cortical inputs to the cerebellum via middle cerebellar peduncle) connectivity is significant when analgesia stage is involved in the statistical contrast suggests that at least part of the difference in pain intensity can depend on the processing of nociceptive inputs performed by cerebellum.

There is abundant literature showing that the anisotropy of the remaining brain areas observed in our study (as corpus callosum, internal capsule, corona radiata, or superior longitudinal fasciculus) seem to be altered in chronic pain (Moayedi et al., 2012; Yoon et al., 2013; Yu et al., 2013; Lieberman et al., 2014). Little evidence is available on the relationship between acute pain and white matter integrity. Using functional DTI (fDTI), a decrease in FA in response to painful stimulation in the contralateral spinothalamic tract has been observed (Lin et al., 2018). This change is specific to the stimulation as the control region does not showed FA alterations. An association between fMRI BOLD response and fractional anisotropy of the midcingulate cortex in response to thermal stimulation has been also observed, so that the functional response was positively correlated with the FA in internal capsule and negatively to that of the cingulum (Warbrick et al., 2016). In the same vein, our results points out that the association of white matter integrity measures and acute pain does not only indicate potential changes in brain structure but that the strength of the association can depend on the actual pain intensity (Liu et al., 2017), even when the white matter integrity is assessed before the pain induction protocol.

Our results have some limitations that are important to take into account. Firstly, sample size can be limited as a consequence of the pain model we have used in our study. However, we have run a power analysis, based on the subjective measures that have the aim to overcome this limitation. Secondly, we have used a subjective pain assessment scale, which cannot discriminate between the different dimensions of pain experience. A more objective measure of pain can produce a different set of results. However, one very important component of pain is how one feels about, that is, the subjective part, which indicates that our results can be interesting in this regard. Thirdly, we have used a b-value of 1,000 s/mm^2^ that will render more difficult to resolve intravoxel crossing fibers (Xie et al., 2015). Further research will be needed in order to disentangle the associations of these dimensions with brain white matter integrity and connectivity. Finally, the cultural and socioeconomic level of our participants is medium-high, which can have an impact on the subjective ratings of pain and on the generality of our results. Further research is needed in order to determine whether the associations we have observed here hold for other cultural and socioeconomic statuses.

Acute pain is associated with white matter integrity, measured by FA, and connectivity, measured by connectometry. FA results suggest that both specific tracts (and number of voxels) associated with pain are a function of the differential level of acute pain. Connectometry results suggest that the white matter tracts (corticospinal tract, medial lemniscus pathway, corpus callosum, middle cerebellar peduncle, and corona radiata) typically involved in pain processing are related to the differences in pain intensity in between the postsurgical stages (anesthesia, pain, analgesia), but the strength of these relationships, as measured by the total count of fibers, seems to depend more on the differences between types of pain relief (anesthesia, analgesia). Specifically, we observed that the difference between the pain stage and the anesthesia stage (the lower difference in subjective pain) embraced much more bilateral tracts than that between pain–analgesia or anesthesia–analgesia (that uniquely involved middle cerebellar peduncle and pontine crossing). Thus, it seems that the number of fibers showing a significant association can depend more on the action mechanisms or the pain-relief effectivity of anesthesia and analgesia than on the action mechanisms of subjective pain.

## Summary Statement

The association of white matter fractional anisotropy and connectivity, measured before the pain stages, with subjective pain depends on the magnitude of the differences in pain scores.

## Data Availability Statement

The original contributions presented in the study are included in the article/supplementary materials, further inquiries can be directed to the corresponding author/s.

## Ethics Statement

The studies involving human participants were reviewed and approved by University of Granada Ethics Committee on Human Research (n° 877). The patients/participants provided their written informed consent to participate in this study.

## Author Contributions

AC and PG-M: conceptualization, methodology, and supervision. AC: data curation and resources. AC, LT-M, and CS-G: formal analysis. AC and LT-M: investigation. AC, PG-M, and LT-M: project administration. AC, PG-M, LT-M, MP-M, CS-G, and FO'V: visualization and writing. All authors contributed to the article and approved the submitted version.

## Conflict of Interest

The authors declare that the research was conducted in the absence of any commercial or financial relationships that could be construed as a potential conflict of interest.
